# Nursing Home Admission during the First Year after Hospitalization – The Contribution of Cognitive Impairment

**DOI:** 10.1371/journal.pone.0086116

**Published:** 2014-01-31

**Authors:** Anne-Sofie Helvik, Randi Helene Skancke, Geir Selbæk, Knut Engedal

**Affiliations:** 1 Department of Public Health and General Practice, Faculty of Medicine, Norwegian University of Science and Technology (NTNU), Trondheim, Norway; 2 St Olav's University Hospital, Trondheim, Norway; 3 Innlandet Hospital Trust, Division Tynset, Tynset, Norway; 4 Centre for Old Age Psychiatric Research, Innlandet Hospital Trust, Ottestad, Norway; 5 Akershus University Hospital, Lørenskog, Norway; 6 The Norwegian Centre for Dementia Research, Oslo University Hospital, Ullevaal, Norway; 7 Faculty of Medicine, University of Oslo, Oslo, Norway; University of Glasgow, United Kingdom

## Abstract

**Objective:**

The importance of cognitive and physical functioning for nursing home admission among medically hospitalized older patients is rarely studied in a one-year follow-up perspective. This study aims to explore the association between patient characteristics and nursing home admission within one year after hospitalization in persons 65 years or more.

**Design:**

A one-year longitudinal study.

**Methods:**

We included 463 (234 women) persons aged 65 years or more from internal medical wards in a rural area of Norway. Cognitive function was assessed using the Mini Mental State Examination; physical and instrumental functional status was assessed using the physical self-maintenance scale and instrumental activities of daily living scale of Lawton and Brody. Comorbidity was measured with the Charlson index. Admission to nursing home within one year (yes versus no) was analyzed using logistic regression analysis and Cox proportional hazard regression analysis.

**Results:**

The mean age of the sample was 80.5 (SD 7.4) years, mean Mini Mental State Examination score was 24.1 (SD 3.8) (maximum score is 30). In adjusted analysis participants with cognitive impairment (a Mini Mental State Examination score <25) or impaired physical functioning at baseline had higher risk of admission to nursing home within one year (OR 3.0, 95%CI 1.5–6.2 and OR 3.5, 95%CI 1.8–9.6, respectively). The time before admission was also associated with cognitive impairment and impaired physical functioning in the adjusted analysis (HR 2.6 95%CI 1.4–4.8 and HR 3.7, 95%CI 1.5–8.9, respectively).

**Conclusion:**

Impaired cognitive and physical functioning increased the risk for nursing home admission within one year after hospitalization. However, putative regressors, such as education and social network were not included in the analysis.

## Introduction

The risk of admission to nursing home after discharge from hospital within a period of one year is increased compared to older persons with no hospitalization [1], [2]. An acute medical illness in older persons causing a hospitalization may increase the risk of cognitive decline [3]. Even so, there are studies reporting cognitive reduction occurring after hospitalization in older patients with a wide variety of diagnoses and treatments [4], [5]. The independent importance of cognitive function at baseline for later nursing home admission among medically hospitalized older persons is rarely studied in the perspective of one year or longer, but community-based studies have shown that more severe cognitive impairment independently increases the risk of nursing home admission [1], [6], [7]. In a recently published study, using a validated screening tool it was reported that the risk of nursing home admission within one year was very high among older community dwelling persons with pronounced cognitive impairment [8].

Three systematic reviews, of studies published between 1977 and 2008, of factors affecting nursing home admission after hospitalization in older patients [9]–[11] have identified 24 studies. Most of the studies were published before 1990, and reviewed factors associated with nursing home admission at discharge or after a short period of time (1 to 6 months). Only four studies had a long-term perspective of a one year follow-up [12–15]. The heterogeneity of assessment tools and outcome measures made it difficult to conclude which health factors predicted nursing home admission [11], but generally it was reported that general medical health, physical functioning, cognitive impairment and psychological health were important factors [9]–[11]. Lately, two studies following previously hospitalized older patients over 12 months have been published [2], [16]. One of them followed acutely hospitalized Dutch older patients and found failure to regain functional status within three months after hospitalization to be important for nursing home admission [2].The second study including patients discharged from internal medicine wards in France, found that a higher frailty index score increased the risk of institutionalization within one year [16]. The index included health indicators such as physical and instrumental functioning, balance or risk of falls, cognitive function and comorbidity in addition to demographic situation [16]. The importance of each health indicator for nursing home admission was, however, difficult to assess in this study because the authors used a single index measure instead of multiple measures.

A few Nordic studies (including studies from Norway, Sweden, Denmark, Finland and Iceland) have focused on the risk factors of nursing home admission among older persons living at home receiving in-home care [17]–[20]. However, as far as we know, no Nordic studies or studies from rural areas have explored the risk factors associated with nursing home admission in a short term or a one year follow-up perspective in previously medically hospitalized older patients. The structure of the rural communities may contribute to additional challenges with health services, transportation and participation in community activities for older adults. The trends in nursing home admission may differ over time [7], [21], as well as being influenced by culture, political priorities, financial support [22], and the available number of nursing home beds [23]. In Norway, nursing home care is a public service and is the responsibility of the municipalities. The municipalities also provide social services and in-home nursing care to their residents. Older persons in need of extensive care that cannot be provided by professional carers are normally admitted to nursing home care. The average age of nursing home residents is about 84 years and 70% are women [24]–[27].

Our aim was to study the association between patient characteristics and nursing home admission within one year after hospitalization among older persons with a particular focus of cognitive impairment assessed during hospitalization. We hypothesized that impaired cognition was associated with an increased risk of nursing home admission.

## Methods

### Ethical statement

The study was approved by the Regional Committee for Medical Research Ethics in South-Eastern Norway (402-06164 1.2006.2106) and the Norwegian Social Science Data Service (14931).

### Design

During a two-year period (1 September 2006 – 30 August 2008) a study was performed including older patients (≥65 years) at a general public hospital in Norway [28], [29]. The hospital serves nine inland municipalities covering an area of 15,000 km^2^ with 25,000 inhabitants, where 4,600 persons are 65 years or older. The participants in the study were followed up one year after inclusion.

### Participants

All patients 65 years or older, living in the region, admitted to the internal medical inpatients service of the Tynset Division of the Innlandet Hospital Trust with an acute medical condition and hospitalized for at least 48 hours were potential participants. Of the 802 available study participants, 318 (40%) were excluded due to: severe cognitive impairment (116 patients) signified by a score of three on the Clinical Dementia Rating Scale (CDR) [30], [31]; severe communication difficulties (25 patients); being in a terminal state or having died before inclusion (47 patients); reduced physical functioning that made completion of the protocol impossible (mainly caused by profound cardiovascular, pulmonary or cancer diagnoses) (106 patients); or, refusal to participate (24 patients) [28], [29]. A total of 484 patients were assessed for inclusion in the study. However, 16 of these patients had been living in a nursing home immediately before the hospital admission and 5 patients died before discharge. Thus, 463 patients entered the study.

### Measures

Cognitive function was assessed by means of the Mini Mental State Examination (MMSE), a 30-point questionnaire [32] where a Mini Mental State Examination score of 25 or higher on the Norwegian version indicates minimal or no cognitive impairment [33]. The Clinical Dementia Rating Scale (CDR) assessed the severity of the dementia and a total score of 3 (range 0–3) indicates severe dementia [31].

Physical health (number of hospitalizations in the previous five years, length of hospital stay, and diagnoses at inclusion in the study) was obtained from medical records or the hospital's administrative systems. Details of co-morbid diseases were collected at inclusion using the Charlson Index [34] and employing Schneeweiss weighting [35].

Level of functioning (Activities of Daily Living - ADL) was measured by the Physical Self-Maintenance Scale (PSMS, score range 6–30) and the Instrumental Activities of Daily Living scale (I-ADL, score range 8–31) [36]. Physical functioning is the outcome of the Physical Self-Maintenance Scale and instrumental functioning is the outcome of the Instrumental Activities of Daily Living scale. Lower scores indicate a higher level of functioning, and having a Physical Self-Maintenance Scale sum score of 6 and an instrumental activities of daily living scale sum score of 8 indicate a normal level of functioning [36]. The fall tendency and vision/hearing functions were self-reported by single items from the population based Health Study of Nord-Trøndelag [37] and Resident Assessment Instrument (RAI-AC) [38], respectively.

Depressive and anxiety symptoms were assessed using the self-report inventory Hospital Anxiety and Depression (HAD) scale. The scale has 14 items assessing depressive and anxiety symptoms (seven items each with a score range of 0–21) [39]. Higher scores indicate more severe symptoms. The cut-off point for having clinically significant depression (HAD-D) or anxiety (HAD-A) was set to ≥8 in each sub-scale [40]. The Hospital Anxiety and Depression scale has been translated into Norwegian and is validated in Norway and used in several studies including some among old adults [29], [41].

Socio-demographic information (living alone or not, smoking habits and residence details) was self-reported using questions from the population-based health studies undertaken in Nord-Trøndelag county [37].

Place of residence (Municipality) was registered according to information in the hospital administrative systems given in the national register.

Time of death. In Norway time of death is registered in the Cause of Death Registry and transferred electronically to the hospital administrative system based on the unique national 11 digit identity number. Thus, information about time of death was collected from the hospital administrative system.

Registration on becoming a permanent nursing home resident within the first year after hospitalization and prior to death was done by use of a “registration card” given to the patients, their families and the caregivers [42]. In addition, we used the registration system in the hospital and other institutions to verify the information given and missing registration cards, and control the time of admission to the nursing home and to make sure that the admission was meant to be permanent.

### Procedure

All the patients aged 65 years or older were invited to participate during their hospital stay just after they had been medically stabilized. The date and time of their inclusion in the study was registered. The patients received written and verbal information about the study, and they subsequently gave their written consent. In case of lacking capacity to consent the patients' next of kin could refuse participation on behalf of the patient. Initially, the Mini Mental State Examination was administered to all potential patients. If the MMSE score was 18 or lower, the CDR was performed. Those with severe dementia (CDR = 3) were excluded. If needed, the patients were given help to read and tick off the self-report questionnaires.

The patients included at baseline were contacted twice; first, after 6 months with a posted letter reminding them about the study, asking for the return of the first 6 months of registrations and with registration cards for the next 6 months. Second, the participants were contacted one year after inclusion (±14 days) and the registration cards were collected at the site. The follow-up contact was performed by the same two registered nurses (one specialized in geriatrics and one in health science) who collected the data at baseline. However, the data collectors did not have access to baseline data, except for the hospitalization period and the inclusion date, during the follow-up data collection. Prior to the start of the study, the nurses completed a two-day course on how to conduct the interview, followed by practicing on a number of healthy subjects.

### Data analysis

Data were analyzed by means of the IBM SPSS, version 19.0 (Chicago, Ill, USA). Descriptive analysis of independent samples was performed with the chi-square statistic or Fisher's Exact Test for categorical variables (depending on the number of cases included). Independent sample *t*-tests/ANOVA or the nonparametric Mann–Whitney test was performed for continuous variables (depending on whether or not the distribution was normal).

Nursing home admission was first analyzed using logistic regression analysis (the ‘Enter’ method) in order to find the risk of nursing home admission within the first year (yes/no). In the initial analysis, age, gender, living alone, smoking, municipality of residence, death within the year (yes/no), number of hospitalizations in the previous five years, elevated comorbidity, Mini Mental State Examination, physical functioning, instrumental functioning, hearing impairment, vision impairment, fall tendency and depression and anxiety (according to HAD) were studied. The level indicating the best situation at baseline (T1) was set as the reference when possible for both continuous and categorical variables. The sum scores at T1 for physical and instrumental functioning, depression and anxiety symptoms were dichotomized because of a non-linear association with nursing home admission within the first year and clinically accepted cut-off scores for the relevant variables were used. The variables presented were either significantly associated or tended to be significantly associated (p<0.1) with the outcome after adjustment for age, gender, municipality of residence and death (yes/no) within the follow-up year. These independent variables were included in the adjusted model. Separate logistic regression models were constructed in order to examine possible interactions between the independent variables. No interactions were found.

Second, the time to nursing home admission within the first year after discharge from hospital was studied by use of Cox proportional hazard regression analysis. This analysis takes into account both the importance of baseline vital status and the time before the event occurs. Censored participants were those dying within a year without nursing home admission. A graphical inspection of the proportionality of the hazard assumption was carried out. Furthermore, we checked whether independent variables were time-dependent for the outcome. The proportional hazard was tenable and no interactions between independent variables were found. The same initial independent variables, the same reference levels and the same assumptions for presentation of the analysis and inclusion in further analyses were used in this assessment method as in the logistic regression analyses.

P-values ≤0.05 were regarded as statistically significant.

## Results

### Sample characteristics

The sample included 229 men and 234 women (see table 1). At baseline the patients' age range was 65–101 years (mean 80.5, SD 7.5 years). Within the one year follow-up period, 85 patients (18.3%) had become nursing home residents, with a variation between 10.7% (lowest) and 28.4% (highest) (p>0.05), see table 2.

**Table 1:** Baseline characteristics of study sample, those admitted to nursing home or not within one year.

		Total		Nursing home admission		Not admitted		p-value
		463	*(100)*	85	*(100)*	378	*(100)*	
**Socio-demographic**								
Women N (%)		234	*(50.5)*	52	*(59.1)*	182	*(48.2)*	* *a*
Age	Mean *(SD)*	80.5	*(7.4)*	84.6	*(6.8)*	79.5	*(7.3)*	**
Living alone	N (%)	231	*(49.9)*	49	*(57.6)*	182	*(48.2)*	
Smoking	N (%)	59	*(12.4)*	8	*(9.4)*	51	*13.5)*	
**Medical information**								
Assisted living before hospitalization								
Nursing care at home	N (%)	156	*(33.7)*	52	*(59.1)*	104	*(27.5)*	** *b*
Residential assistance a	N (%)	57	*(12.3)*	8	*(9.4)*	49	*(13.0)*	
No assistance or care	N (%)	250	*(54.0)*	25	*(29.4)*	225	*(59.5)*	
Previous hospitalizations in last 5 years	Mean *(SD)*	1.3	*(1.1)*	1.5	*(1.1)*	1.3	*(1.1)*	
Actual hospitalization (days)								
Duration	Mean *(SD)*	6.5	*(5.3)*	8.9	*(6.6)*	5.9	*(4.8)*	
Duration before inclusion	Mean *(SD)*	4.3	*(3.6)*	5.5	*(5.1)*	4.1	*(3.1)*	
Charlson Index	Mean *(SD)*	2.1	*(2.0)*	2.7	*(2.3)*	2.0	*(1.9)*	**
Principal diagnosis on admittance								
Cardiovascular disease	N (%)	128	*(27.6)*	25	*(29.4)*	103	*(27.3)*	
Pulmonary disease	N (%)	98	*(21.2)*	11	*(12.9)*	87	*(23.0)*	
Number of medicaments	Mean *(SD)*	5.9	*(3.1)*	6.3	*(2.9)*	5.8	*(3.2)*	
**Impairment**								
MMSE	Mean *(SD)*	24.1	*(*3.8)	21.6	*(3.5)*	24.6	*(3.6)*	**
PSMS	Mean *(SD)*	8.7	(3.2)	11.4	*(4.0)*	8.1	*(2.6)*	**
I-ADL	Mean *(SD)*	9.6	*(3.6)*	11.7	*(3.9)*	9.2	*(3.3)*	**
Had falls in the previous year	N (%)	126	*(27.2)*	31	*(36.5)*	95	*(25.1)*	* *c*
Impaired hearing	N (%)	178	*(38.4)*	33	*(38.8)*	145	*(38.4)*	
Impaired reading vision	N (%)	98	*(21.2)*	20	*(23.5)*	78	*(20.6)*	
**Emotional situation**								
Prevalence of depressive symptoms (HAD-D ≥8)	N (%)	43	*(9.3)*	9	*(10.6)*	34	*(9.0)*	
Prevalence of anxiety symptoms (HAD-A ≥8)	N (%)	42	*(9.1)*	9	*(10.6)*	33	*(8.7)*	

MMSE = Mini Mental State Examination, PSMS = Personal functioning, I-ADL = Instrumental functioning.

HAD-D = The depression subscale of the Hospital Anxiety and Depression scale with, HAD-A = The anxiety subscale of the Hospital Anxiety and Depression scale.

a Assistance with cleaning, making food, doing groceries etc.

* = p< 0.05,

**p<0.01.

*a* = Pearson Chi-Square 4.712 (1 df) p = 0.028.

*b* = Pearson Chi-Square 35. 049 (2 df) p<0.001.

*c* = Pearson Chi-Square 4.443 (1 df) p = 0.035.

*d* = Pearson Chi-Square 67.579 (1df) p<0.001.

**Table 2:** Nursing home admission by the municipality of residence.

Municipality no	Total at T1		Nursing home admission within a year		Proportion of admission to nursing home by number of participants in each municipalities		
	N (%)		N (%)		%		
	463	*(100)*	85	*(100)*			
1	28	*(6.0)*	5	*(5.9)*	*17.9*		
2	38	*(8.2)*	7	*(8.2)*	*18.4*		
3	58	*(12.5)*	7	*(8.2)*	*12.1*		
4	44	*(9.5)*	10	*(11.8)*	*22.7*		
5	109	*(23.5*	31	*(36.5)*	*28.4*		
6	33	*(7.1)*	4	*(4.7)*	*12.1*		
7	28	*(6.0)*	3	*(3.5)*	*10.7*		
8	100	*(21.6)*	15	*(17.6)*	*15.0*		
9	25	*(5.4)*	3	*(3.5)*	*12.0*		

In bivariate analyses the following characteristics were related to nursing home admission: being a woman, assisted living before hospitalization, falls in the year prior to hospitalization, being older, comorbidity, poorer cognitive function, and impairment in physical and instrumental functioning.

### Factors associated with nursing home admission within 12 months after discharge from the hospital - multivariate analysis

The odds for becoming a nursing home resident within 12 months increased with increasing age after adjustment for gender, municipality of residence and death within the follow-up year (OR = 1.09, 95%CI = 1.05–1.14).The length of time before nursing home admission was also associated with increasing age after adjustment for gender, municipality and death within the follow-up year (HR = 1.12, 95% CI = 1.08–1.15).

In the logistic regression model, cognitive impairment and impaired physical functioning at baseline were independently associated with increased risk of nursing home admission within a year (OR = 3.02, 95%CI = 1.47–6.19 and OR = 3.51, 95%CI = 1.79–9.63, respectively) when adjusted for age, gender, municipality, death within a year, any falls in previous 12 months before hospitalization, impaired instrumental functioning and comorbidity (elevated Charlson index) at baseline (Table 3). In the adjusted Cox proportional hazard regression analysis, the length of time before nursing home admission was found to be associated with impaired cognitive and physical functioning (HR = 2.65, 95%CI = 1.45–4.83 and HR = 3.66, 95%CI = 1.51–8.86, respectively) (Table 4).

**Table 3:** Associations with nursing home admission versus not within the follow-up year (N = 463).

At T1	OR_1_ 95% CI	OR_2_ 95% CI
MMSE <25	**4.089 (2.0651–8.095)**	**3.017 (1.470–6.191)**
PSMS >6	**5.323 (2.088–13.565)**	**3.510 (1. 792–9.628)**
I-ADL >8	**1.994(1.063–3.739)**	1.151 (0.579–2.286)
Have fallen the past year before T1	**1.889 (1.052–3.393)**	1. 565 (0. 852**–**2.878)
Elevated Charlson Index	**2.247 (1.050–4.805)**	1.938 (0.876–4.286)
−2 Log likelihood/Nagelkerke R Square in %		307.613/40.7

MMSE = Mini Mental State Examination, PSMS = Personal functioning, IADL = Instrumental functioning.

OR = odds ratio, CI = Confidence intervals.

_1_The variables presented in the models are adjusted for age, gender, municipality of residence and death within a year.

_2_The variables presented in the model are adjusted for age, gender, municipality of residence, death within a year and each other.

Bold text is significant associations.

T1 = baseline.

**Table 4:** Hazard ratio associations before nursing home admission during the follow-up year (N = 463).

At T1	HR_1_ 95% CI	HR_2_ 95% CI
MMSE <25	**3.510 (1.958–6.290)**	**2.645 (1.447–4.834)**
PSMS >6	**5.366 (2.276–12.653)**	**3.655 (1.508–8.860)**
I-ADL >8	**2.134 (1.237–3.681)**	1.218 (0.689–2.154)
Have fallen the past year before T1	1.359 (0.856–2.157)	
Elevated Charlson Index	**1.991 (1.067–3.716)**	1.682 (0.894–3.164)
−2 Log likelihood/Chi-square(df)		885.791/91.348(14)

MMSE = Mini Mental State Examination, PSMS = Personal functioning, IADL = Instrumental functioning.

HR = Hazard ratio, CI = Confidence intervals.

_1_The variables presented in the models are adjusted for age, gender and municipality of residence.

_2_ The variables presented in the model are adjusted for age, gender, municipality of residence and each other.

Bold text is significant associations.

T1 = baseline.

## Discussion

To the best of our knowledge, this is the first follow-up study in the Nordic countries of elderly medically hospitalized adults to study factors associated with nursing home admission within the first year after hospitalization. The study was performed among older patients in rural municipalities. Independent of the participants' age, gender, municipality of residence, the tendency to fall before hospitalization, impaired instrumental and physical functioning and comorbidity at baseline, or death within a year, the risk of nursing home admission was increased in patients with cognitive impairment at baseline. In addition, the length of time before nursing home admission was independently associated with impaired cognitive functioning and physical functioning.

The financial systems, politics, culture, and living conditions may influence the risk factors of importance for nursing home admission in a country [7], [21]–[23], [43]. Thus, risk factors of nursing home admission after hospitalization need to be studied in their own culture and setting if they are to be relevant. In the present study, the mean age of the participants who were admitted to a nursing home within a year after the hospitalization was about the same as the average of nursing home residents in Norway [24]–[27]. Of the total sample 18% were admitted to nursing home within one year. There seems to be some variation between the municipalities (11% to 28%, with the municipality with the highest number of participants having the highest proportion of nursing home admissions. This is probably due to local political priorities. The present study was a single hospital site study and the results may not be generalizable to other parts of Norway. The previously hospitalized older persons with impaired cognitive function were more likely to become nursing home residents than those without impaired cognitive function. This is the case even if health care planners and older persons with a care need seem to prefer in-home nursing prior to nursing home admission [44]. The reason for this may be related to the in-home nursing staff's lack of knowledge about how to handle cognitively impaired older persons in their own homes, or maybe due to lack of personnel resources. In general, persons with cognitive impairment may need repeated daily visits and continuity in the care by involving a restricted number of educated personnel in order to secure adequate in-home care. An additional limiting factor of in-home nursing care in rural areas may be long distances to the persons in need of care. Both impaired cognitive and physical functioning increased the risk for nursing home admission and decreased the time until admission. These results were in line with previous studies using a one year perspective of nursing home admission after hospitalization, which have focused on the importance of dementia [12], [14], impaired physical functioning [2], [13] or combining several health and disability indicators in an index [15], [16]. In our study, the presence of a certain threshold of health difficulties in terms of physical and cognitive impairment increased the future risk of nursing home admission substantially, as was found in a meta-analysis of nursing home admission among older persons in the community [1] and in a recent study of nursing home admission within one year among older community dwelling persons in Canada [8].

The study has limitations. First, the Mini Mental State Examination is mainly a screening tool for dementia and delirium and is a fairly crude way to assess cognitive function, even if, it is widely used in clinical practice. The inventory may underestimate the cognitive impairment and the Mini Mental State Examination cut-off scores for normal cognitive function in older persons are debatable [45]. A minimal cognitive impairment and even dementia may exist even if the Mini Mental State Examination sum-score is not significantly reduced (<25) [46]–[48]. In previous studies, the cut-off point for normal cognitive function based on assessments with the Mini Mental State Examination has often been set, as was ours, to a score ≥25 [49].

Second, the Mini Mental State Examination sum-score was assessed during the hospitalization. We are aware that the cognitive function at that stage may be affected by the ongoing disease and hospitalization and may be reasoned in delirium [50]. An alternative approach would have been to use an assessment prior to the hospitalization, but no pre-hospitalization score was available. In the present study, all baseline assessments were performed when the possible participants' health situation had been stabilized, but several options exist; some baseline assessments have been performed within 24 hours after hospitalization, and others at discharge [10], [12].

Third, even if depression is quite common in hospitalized older adults [29] few of the participants in our sample had clinical signs of depression at T1. The statistical power to explore the relation between existing depressive symptoms and nursing home admission within one year after hospitalization was restricted. An association between depressive symptoms and nursing home admission has been reported in a one-year follow-up study of previously medically hospitalized patients [14] and in some studies of community based older persons [51], [52], but was not reported in the meta analysis of the community dwelling elderly in the USA [1]. Lastly, we were not able to adjust for education, financial situation or social network, all variables that may potentially be relevant in studies of nursing home admission [11], [14].

We believe that the research assistants' knowledge of the baseline results did not influence the documentation of long-term nursing home admission (yes/no), eventually time for such admission or time of death during follow-up. In order to minimize the risk of ascertainment bias the completed and controlled baseline dataset thereafter was handled by others than the research nurses.

Some studies of nursing home admission on discharge from hospital or during the follow-up period of one year after hospitalization have used multiple assessments of health indicators, while others have used indexes based on several health assumptions [9]. We see it as a strength to have used multiple instruments, which has made it possible to study the importance of each health indicator. However, use of multiple measures in a clinical practice is time consuming and tiring for the patient. Thus, an index that includes the important health indicators may be helpful in clinical practice in order to identify those in need of intervention in order to avoid or postpone nursing home admission. Several indexes exist [16], but have shown poor agreement [16] and none of them have been tested in the Nordic countries yet.

## Conclusion

Impaired cognitive and physical function increased the risk for nursing home admission within one year after hospitalization in a Norwegian rural district. However, putative regressors, such as education and social network were not included in the analysis.
